# Enhancing Radicular Defense: The Impact of Cross-Linking Agents on Antibiofilm Activity and Collagen Integrity With Bioceramic Sealers

**DOI:** 10.7759/cureus.89815

**Published:** 2025-08-11

**Authors:** Sevitha Thummala, Surender L.R., Naresh Kumar K, Sravani Nirmala, Narender Reddy, Kavuri Jyostna

**Affiliations:** 1 Conservative Dentistry and Endodontics, SVS (Sri Venkata Sai) Institute of Dental Sciences, Mahabubnagar, IND

**Keywords:** antibiofilm, bioceramic sealer, chlorhexidine, collagen integrity, cross-linking agents, curcumin, e. faecalis, hydroxyproline, photodynamic therapy, root canal treatment

## Abstract

Background: Root canal treatment requires thorough chemomechanical debridement and three-dimensional obturation for a fluid-tight seal. However, complete eradication of microbial biofilms is difficult due to the complexities of root canal anatomy, limiting irrigant penetration. Photodynamic therapy plays a key role in enhancing antimicrobial efficacy. Bioceramic sealers are commonly used due to their biocompatibility, antibacterial properties, and bioactivity, though their high alkalinity can damage exposed collagen, leading to tooth fracture. Cross-linking of collagen can slow biodegradation and improve dentin strength. Medicinal plants, like *Curcuma longa*, offer antimicrobial and immune-modulatory properties, with its extract curcumin also serving as a photosensitizer and cross-linking agent. The study explores curcumin's ability to combat *Enterococcus faecalis* biofilms and improve collagen matrix integrity in root dentin.

Aim and objective: This study aimed to examine the antibiofilm efficacy of collagen cross‑linking agents and their effects on the integrity of the radicular collagen matrix.

Methodology: Thirty extracted single-rooted human teeth were decoronated to standardize the root length to 12 mm and then prepared using ProTaper rotary files (Dentsply Maillefer, Ballaigues, Switzerland) up to size F3. The specimens were randomly divided into three groups based on the irrigant used (curcumin, chlorhexidine, and saline). Each group was further subdivided based on the evaluation method. In one set of samples, *Enterococcus faecalis* was inoculated into the canals and incubated for 21 days. Following disinfection with the respective irrigant photoactivated with a diode laser, bacterial quantification was performed using real-time polymerase chain reaction. Another set of samples was obturated after surface treatment with the same irrigants and stored in artificial saliva. The release of hydroxyproline, an indicator of collagen degradation, was measured spectrophotometrically after 21 days. Data were statistically analyzed using ANOVA and Tukey’s post hoc test with a significance level set at p < 0.05.

Results: Curcumin with light activation (Group 1A) demonstrated the highest cycle threshold values (38.20 ± 1.34), indicating superior bacterial reduction, followed by chlorhexidine (25.06 ± 3.11) and saline. *E. faecalis* counts were lowest in Group 1A (210.60 ± 46.11). Hydroxyproline release was minimal in curcumin-treated samples (0.04 ± 0.02), suggesting better collagen preservation, while the control showed the highest release (1.18 ± 0.28). Statistical analysis confirmed significant differences among groups (p < 0.05).

Conclusion: Curcumin, particularly when photoactivated, exhibits potent antibiofilm effects and protects collagen in root dentin, making it a promising adjunct in endodontic therapy.

## Introduction

The fundamental principle of root canal therapy is effective infection control, which focuses on eliminating existing microbial contamination and ensuring that the root canal system remains protected from future reinfection [1]. Recurrent infections in root canal-treated teeth continue to occur despite adherence to thorough treatment protocols, primarily due to the persistence of *Enterococcus faecalis*, a Gram-positive facultative anaerobe known for its ability to deeply invade dentinal tubules [2,3].

*E. faecalis* not only forms dense colonies on canal walls but also develops biofilms by utilizing periodontal ligament fluid, which shields it from host immune responses and disinfecting solutions [4]. It possesses several virulence factors, including quorum sensing, adhesins, capsular polysaccharide, and collagen-binding protein, all of which contribute to its antibiotic resistance and persistence within the root canal microenvironment [5]. To address this challenge, clinicians are adopting advanced techniques such as light-induced activation of canal irrigating solutions, along with meticulous instrumentation to overcome the anatomical complexities of the canal [6]. Additionally, photodynamic activation of irrigants induces the generation of reactive oxygen species, rendering them bactericidal [7].

Beyond chemo-mechanical debridement, it is essential to seal the canal from the surrounding microenvironment through thorough three-dimensional obturation. Various sealers are used to aid the obturating material in achieving an adequate seal. Currently, bioceramic sealers are highly preferred due to their biocompatibility, bioactivity, water sorption, and antibacterial properties. However, their elevated alkalinity has been associated with collagen degradation, dentin breakdown, and an increased risk of tooth fracture [8].

Notably, collagen cross-linking, a novel technique, has proven beneficial in slowing the rate of biodegradation and enhancing the mechanical properties of collagen. Several agents, including riboflavin, proanthocyanidin, chlorhexidine (CHX), genipin, and green tea, can promote intra- and intermolecular cross-links [9,10]. In the present study, curcumin was selected due to its multiple advantageous properties, making it highly suitable for endodontic applications.

Curcumin, a natural phenolic compound derived from *Curcuma longa* (turmeric), a well-known Indian spice, has well-documented analgesic and anti-inflammatory effects. In addition, it exhibits strong antimicrobial activity and has been shown to inhibit matrix metalloproteinases (MMPs), potentially enhancing the collagen matrix integrity of radicular dentin [11].

Given its promising properties, this study aimed to evaluate the antibiofilm efficacy of curcumin as a collagen cross-linking agent and its impact on the structural integrity of the radicular collagen matrix. The null hypothesis proposed that curcumin irrigant would neither eradicate *E. faecalis* nor inhibit biofilm formation, nor improve the integrity of the radicular collagen matrix, nor reduce hydroxyproline release.

This article was previously presented as a paper at the 24th IACDE National PG Convention, held on February 29th, 2024, in Chennai, Tamil Nadu.

## Materials and methods

This in vitro study was conducted in the Department of Conservative Dentistry and Endodontics, SVS (Sri Venkata Sai) Institute of Dental Sciences, Mahabubnagar, Telangana, India.

Thirty extracted single-rooted human permanent teeth were collected and stored in distilled water. Inclusion criteria included mature, fully developed single-rooted teeth with intact roots, free from caries, fractures, or prior endodontic treatment. Exclusion criteria comprised teeth with open apices, signs of internal or external resorption, anatomical variations (e.g., curved roots and extra canals), or visible cracks/fractures. All teeth were decoronated at the cementoenamel junction (CEJ) using a diamond disc (Mani Inc., Utsunomiya, Japan), standardizing the root length to 12 mm. The specimens were sterilized in an autoclave (Confident Dental Equipments Ltd., Bengaluru, India) at 121°C for 20 minutes. Canal patency was confirmed with a size 10 K-file (Mani Inc., Utsunomiya, Japan), and working length was established 0.5 mm short of the apex. Initial preparation was done up to size 20 K-file (Mani Inc., Utsunomiya, Japan), followed by complete instrumentation using the ProTaper Gold rotary system (Dentsply Maillefer, Ballaigues, Switzerland) up to F3 using the crown-down technique. During instrumentation, canals were irrigated with 1 mL each of 5.25% sodium hypochlorite (NaOCl)(Prime Dental Products Pvt. Ltd., Thane, India), 17% ethylenediaminetetraacetic acid (EDTA) (Prime Dental Products Pvt. Ltd., Thane, India), and 0.9% normal saline (Aculife Healthcare Pvt. Ltd., Ahmedabad, India). After instrumentation, specimens were cleaned in distilled water using an ultrasonic bath (Oscar Ultrasonics Pvt. Ltd., Mumbai, India) for 30 minutes. The roots were dried, coated externally with clear nail varnish, and sterilized again in an autoclave at 121°C and 15 psi for 30 minutes.

This study was approved by the Institutional Ethics Committee of SVS Dental College, Mahabubnagar (Approval No.: SVSIDS/CONS/6/2023).

Preparation of curcumin irrigant

**Figure 1:**
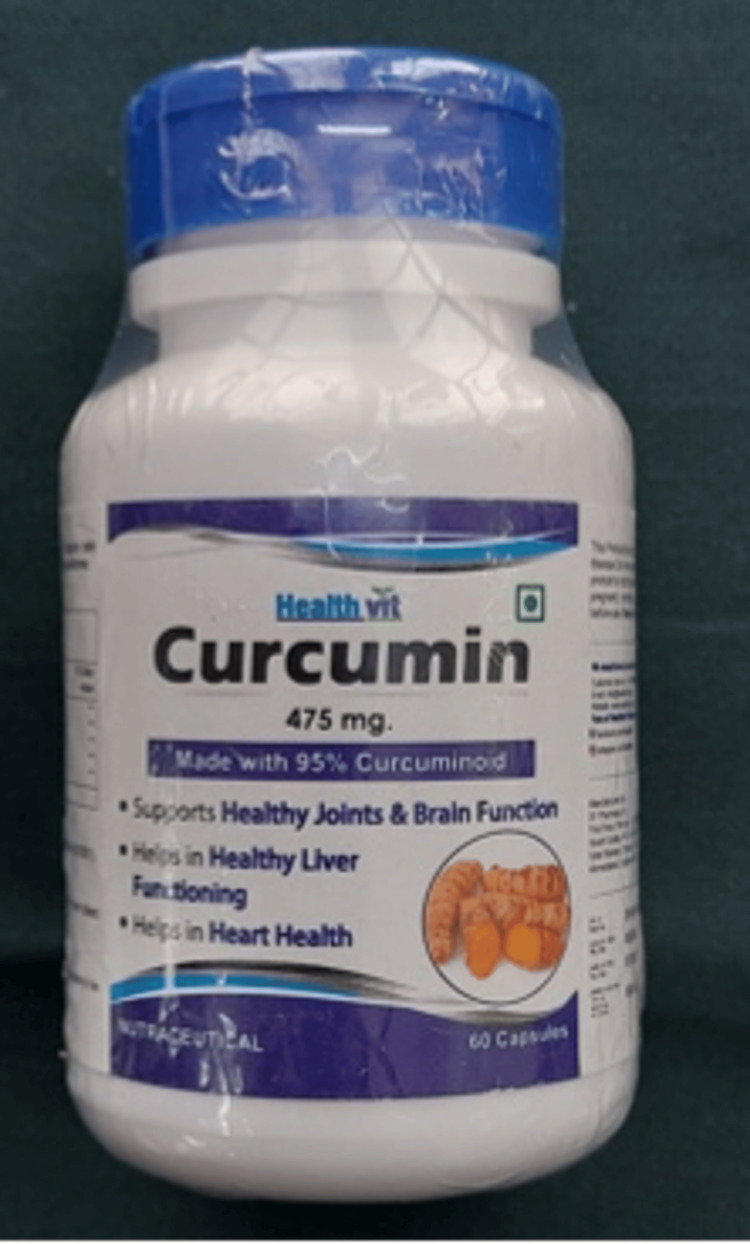
Curcumin.

Pure curcumin powder in capsule form (West Coast Pharmaceutical Works Ltd., Ahmedabad, India) was used (Figure 1).

**Figure 2:**
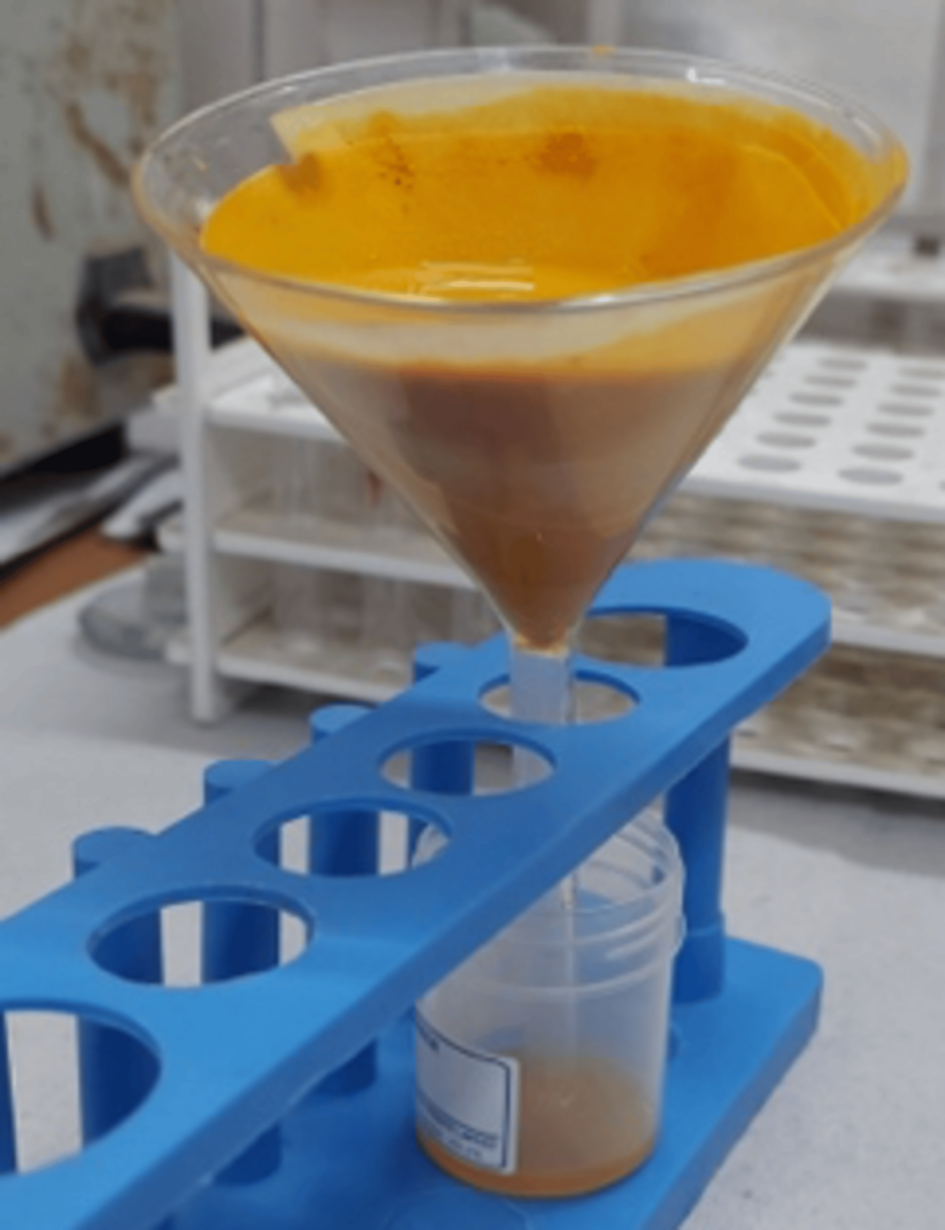
Filtering the solution through Whatman filter paper.

Three grams of curcumin were weighed using an electronic balance (Citizen Scales Pvt. Ltd., Mumbai, India) and mixed with 15 mL of distilled water. The mixture was heated to 100°C on a hot plate until a homogeneous solution formed, then allowed to cool. It was filtered through Whatman filter paper (Cytiva, Little Chalfont, UK) and stored in screw cap test tubes (Borosil Ltd., Mumbai, India) at 4°C (Figure 2). Before use, the solution was brought to room temperature.

Study methodology

The 30 teeth were randomly divided into three main groups (Group 1, Group 2, and Group 3), each with two subgroups (subgroups 1A, 1B, 2A, 2B, 3A, and 3B). Group 1A: curcumin irrigant activated using diode laser (Epic X, Biolase Inc., Foothill Ranch, CA, USA); Group 1B: surface treatment with curcumin; Group 2A: chlorhexidine irrigant (Zodenta, Indoco Remedies Ltd., Mumbai, India) activated using blue light; Group 2B: surface treatment with chlorhexidine; Group 3A: saline irrigant activated using blue light; Group 3B: surface treatment with saline.

Bacterial inoculation

**Figure 3:**
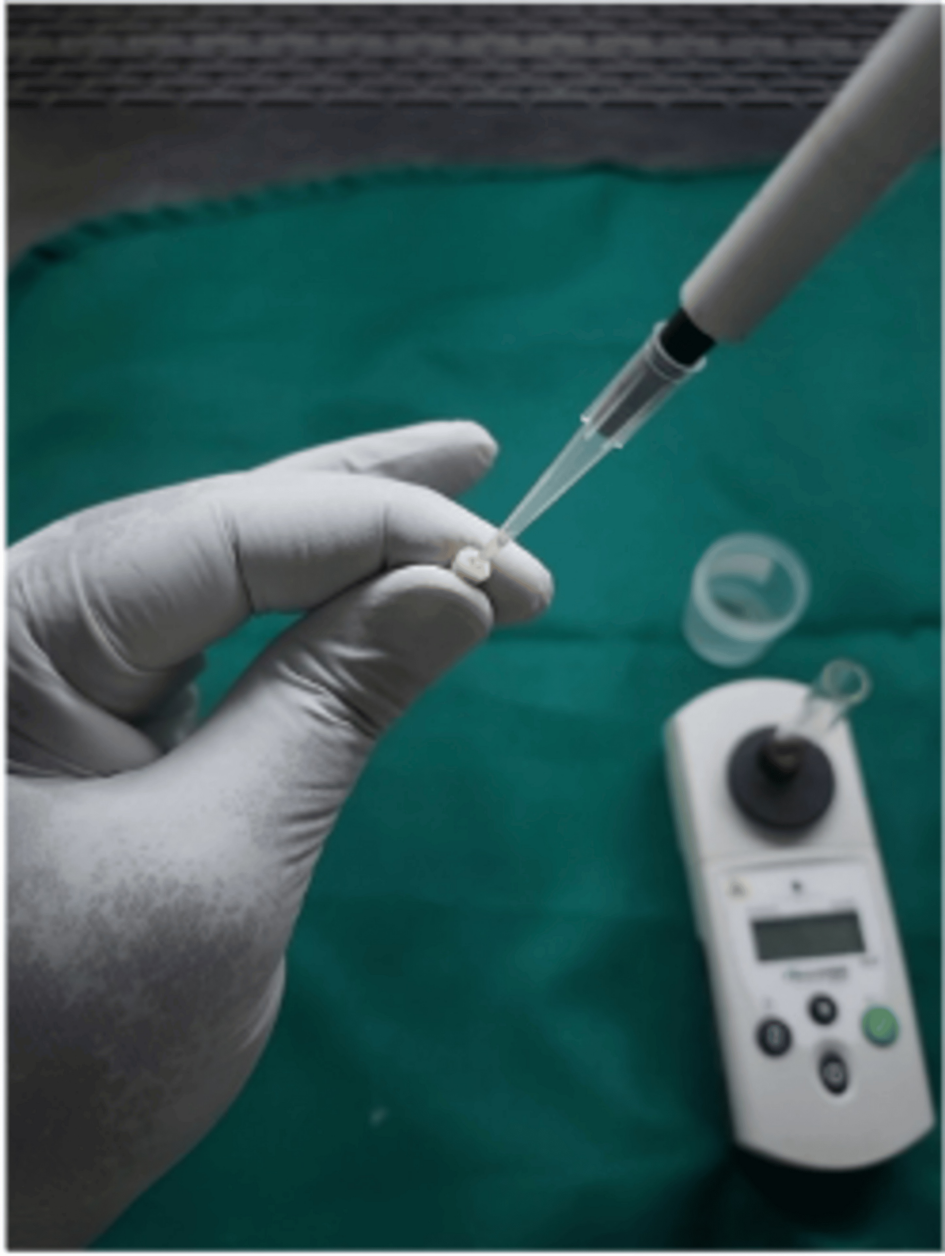
Bacterial inoculation.

All teeth in subgroup A were inoculated with *Enterococcus faecalis* (ATCC strain 29212), prepared in the Department of Microbiology, SVS Institute of Medical Sciences. Apices were sealed with modeling wax (MAARC Dental Products, Satara, India), and 5 µL of bacterial suspension was introduced using a micropipette (Thermo Fisher Scientific Inc., Waltham, MA, USA) under aseptic conditions (Figure 3). Coronal access was sealed with wax, and specimens were incubated (NSW India Ltd., Ambala, India) at 37°C for 21 days. Fresh bacterial suspension was replenished every third day.

Irrigant placement and activation

**Figure 4:**
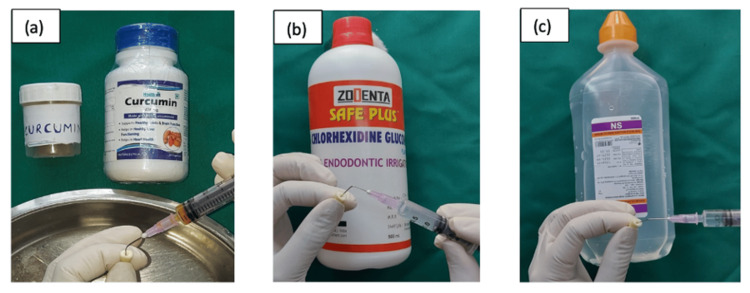
Placement of irrigants.

After incubation, canals were irrigated as per group designation (curcumin, chlorhexidine, or saline) (Figure 4).

**Figure 5:**
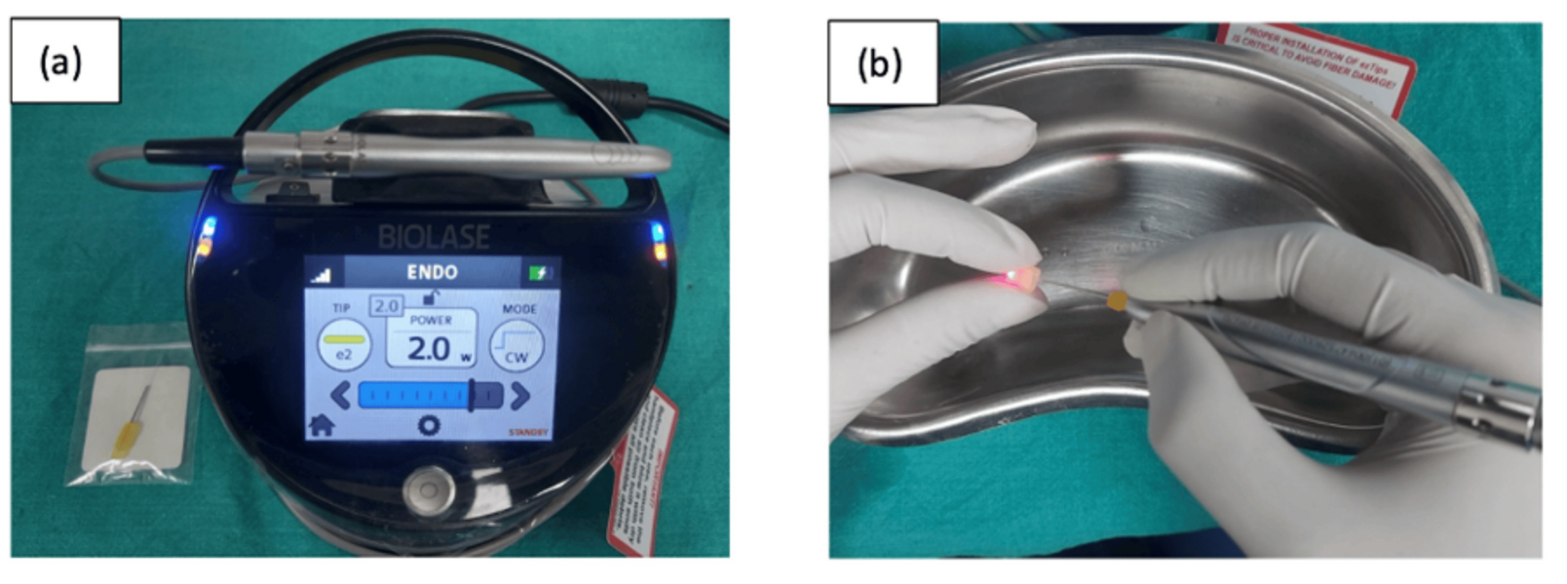
(a) Diode laser (Biolase Epic X). (b) Irrigant activation with laser.

Irrigants were activated with a diode laser (940 nm) (Epic X, Biolase Inc., Foothill Ranch, CA, USA) using a 200 μm fiber-optic tip. The tip was inserted 1 mm short of the apex and moved at 2 mm/s for five seconds. This cycle was repeated four times at 10-second intervals (Figure 5).

**Figure 6:**
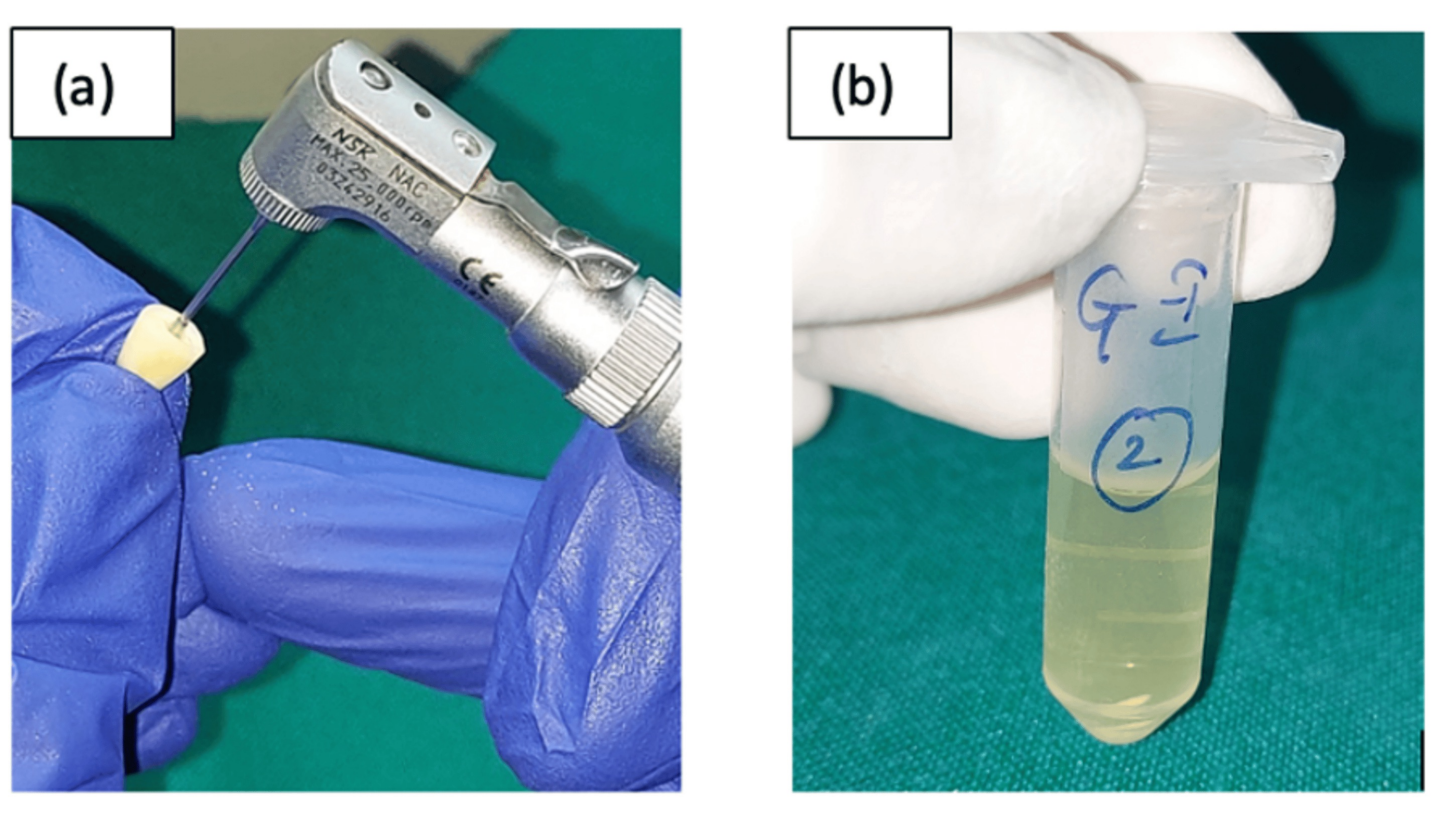
(a) Collection of dentin chips. (b) Collected dentinal chips in sterile Eppendorf plastic tubes.

To assess bacterial presence, dentin chips were collected along the canal length using Gates Glidden drills (sizes 4 and 5) (Mani Inc., Utsunomiya, Japan) and transferred to sterile Eppendorf tubes (Helini Biomolecules, Chennai, India) containing brain heart infusion broth. Samples were incubated at 37°C for 24 hours (Figure 6).

Preparation of purified DNA

**Figure 7:**
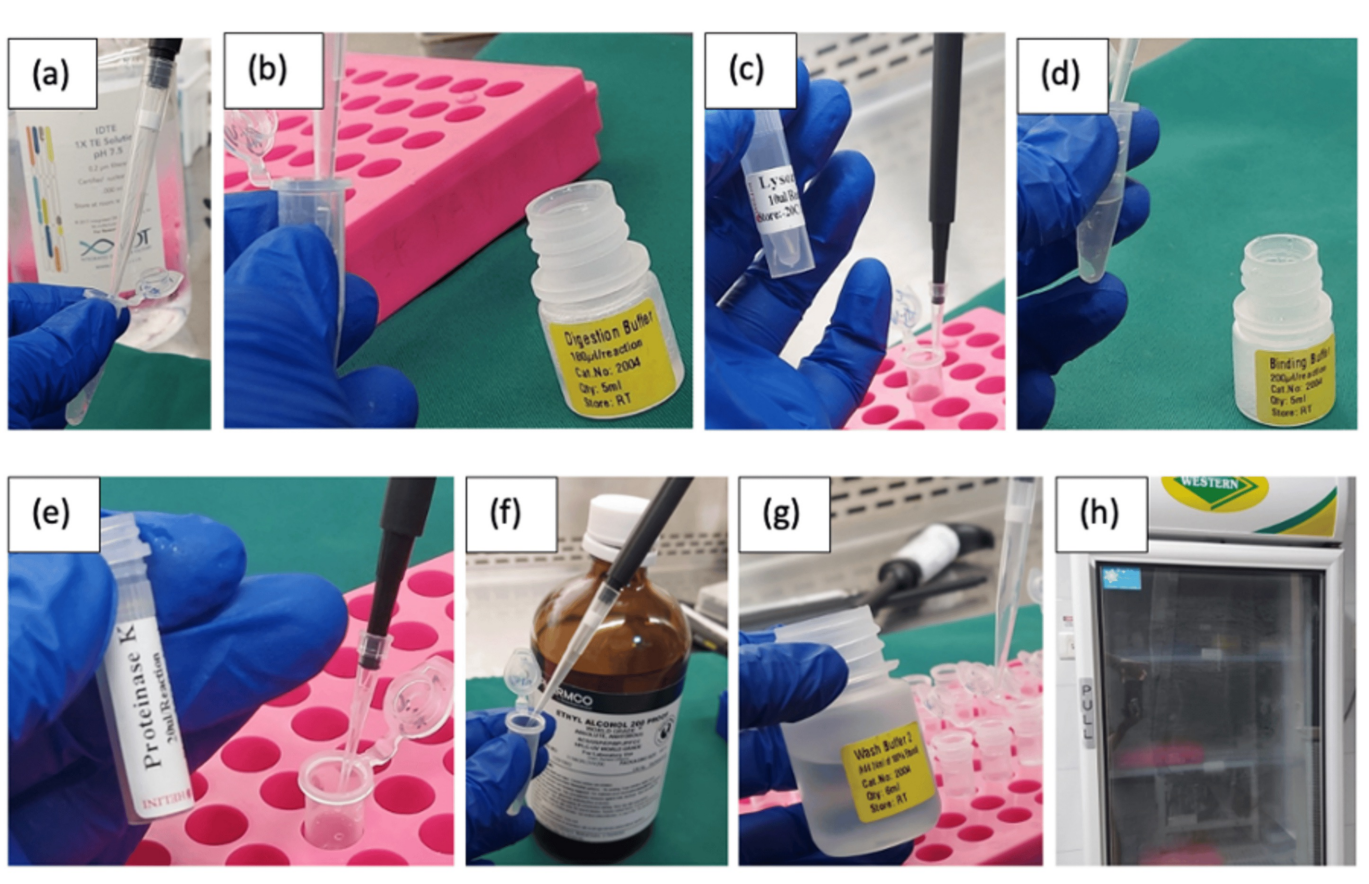
(a) 1 ml of ethylenediaminetetraacetic acid (EDTA) was added. (b) 180 μl of digestion buffer was added. (c) 20 μl of lysozyme was added. (d) 200 μl of binding buffer was added. (e) 20 μl of proteinase K was added. (f) Ethanol was added. (g) 500 μl of wash buffer was added. (h) Purified DNA was stored at -20°C.

Following incubation, the samples were centrifuged (Eppendorf AG, Hamburg, Germany), and 1 mL EDTA was added. Samples were treated sequentially with digestion buffer, lysozyme, proteinase K, and binding buffer (Helini Biomolecules, Chennai, India), incubated at specified temperatures. Ethanol (Pharmco, Brookfield, CT, USA) was added, and the mixture was passed through a Purefast® DNA extraction spin column kit (Helini Biomolecules, Chennai, India) for DNA binding and washing. Purified DNA was eluted and stored at -20°C (Figure 7).

Polymerase chain reaction (PCR) setup

**Figure 8:**
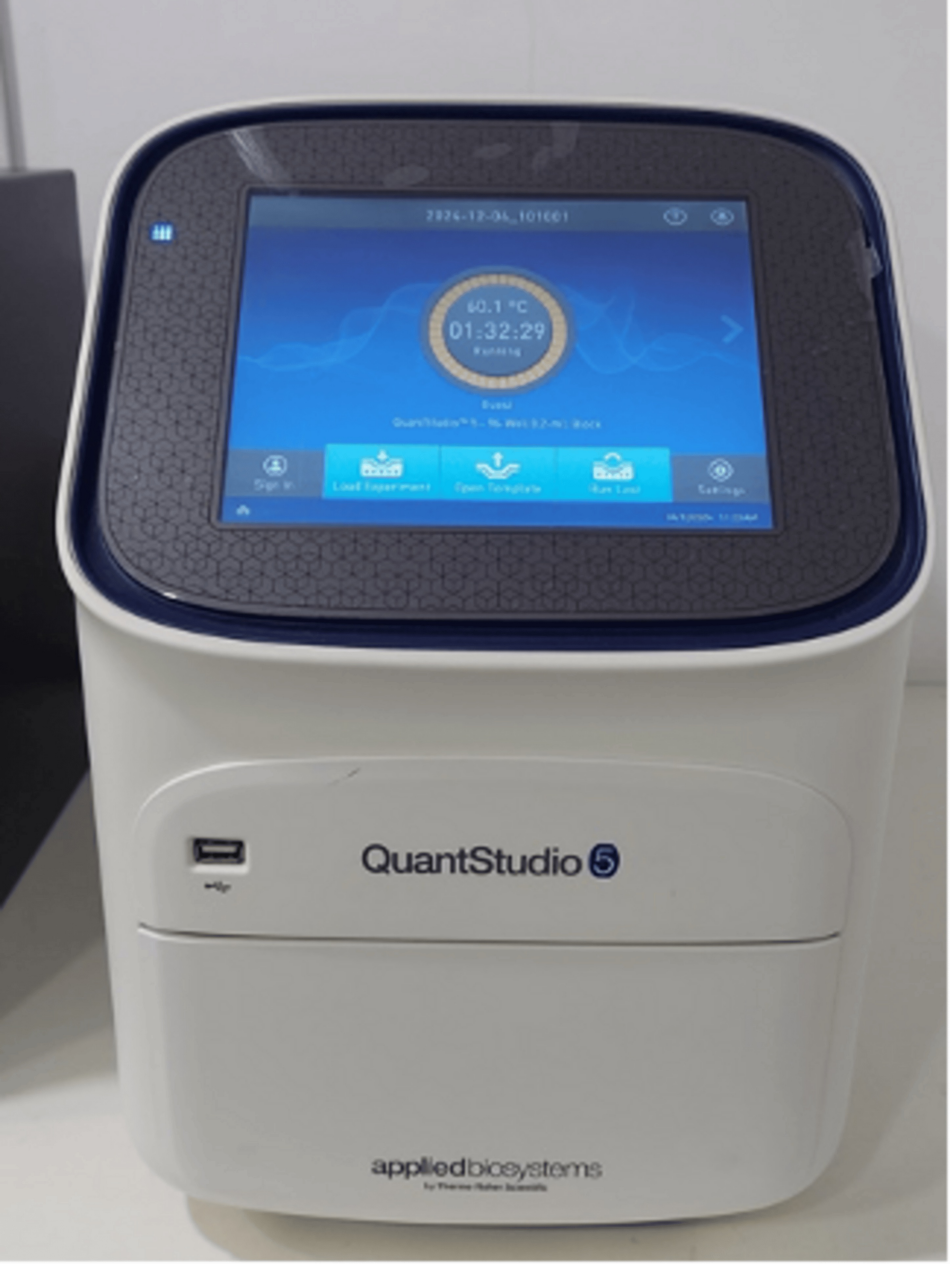
Thermo QuantStudio 5 real-time polymerase chain reaction system.

Purified DNA was mixed with Probe PCR Master Mix, *E. faecalis* PP Mix, and PCR-grade water (Helini Biomolecules, Chennai, India). Negative and positive controls (10 μL each) were included. PCR was performed using a thermocycler (QuantStudio™ 5, Thermo Fisher Scientific Inc., Waltham, MA, USA) to amplify *E. faecalis* DNA (Figure 8).

**Figure 9:**
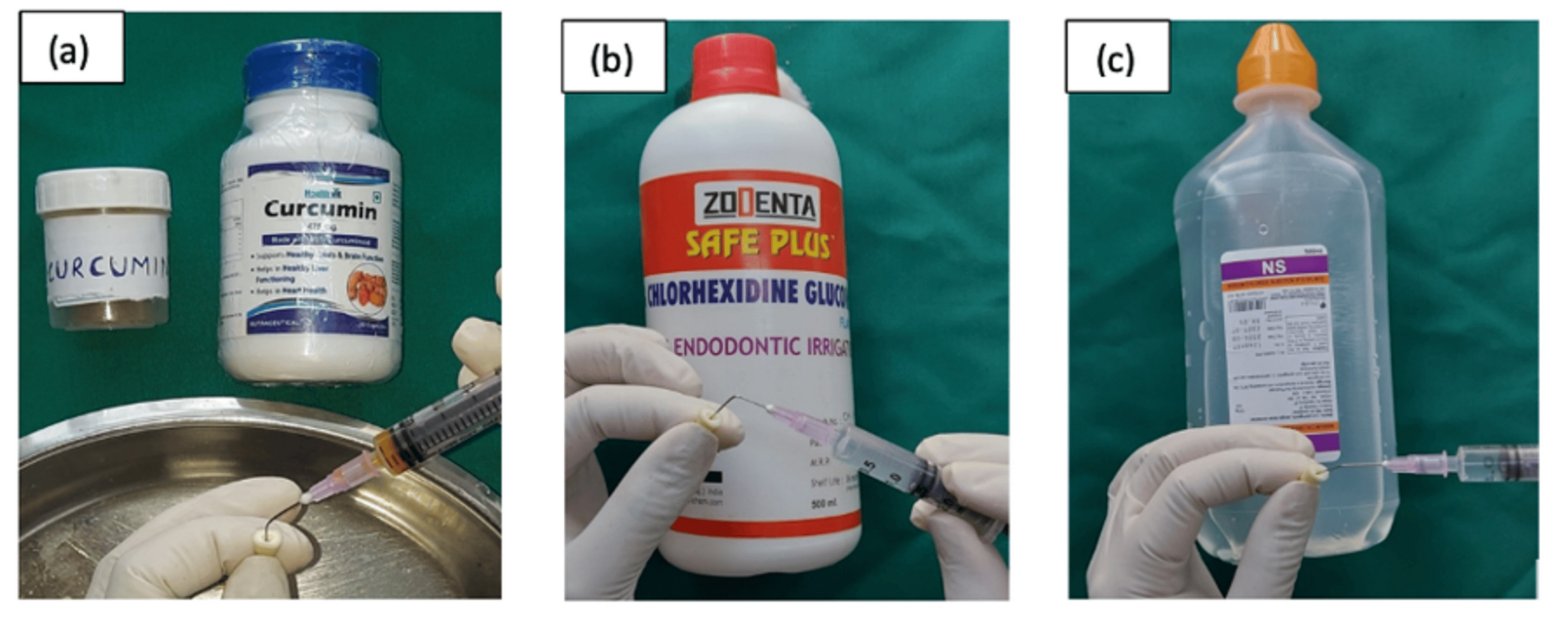
Surface treatment with (a) curcumin, (b) chlorhexidine, and (c) saline.

In subgroup B, following biomechanical preparation, the canals were surface-treated as follows: curcumin (Group 1B), chlorhexidine (Group 2B), and saline (Group 3B) for one minute (Figure 9).

**Figure 10:**
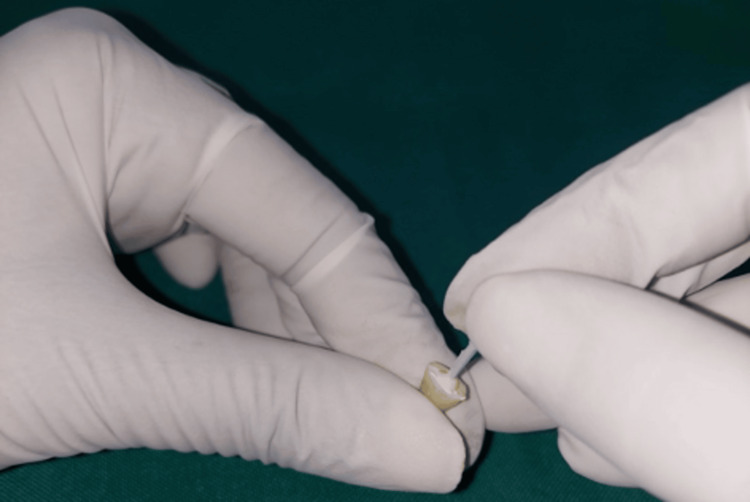
Obturated with gutta-percha and bioceramic sealer.

After drying with paper points (Dentsply Maillefer, Ballaigues, Switzerland), canals were obturated with gutta-percha (Dentsply Maillefer, Ballaigues, Switzerland) and a bioceramic sealer (Prime Dental Products Pvt. Ltd., Thane, India) (Figure 10).

**Figure 11:**
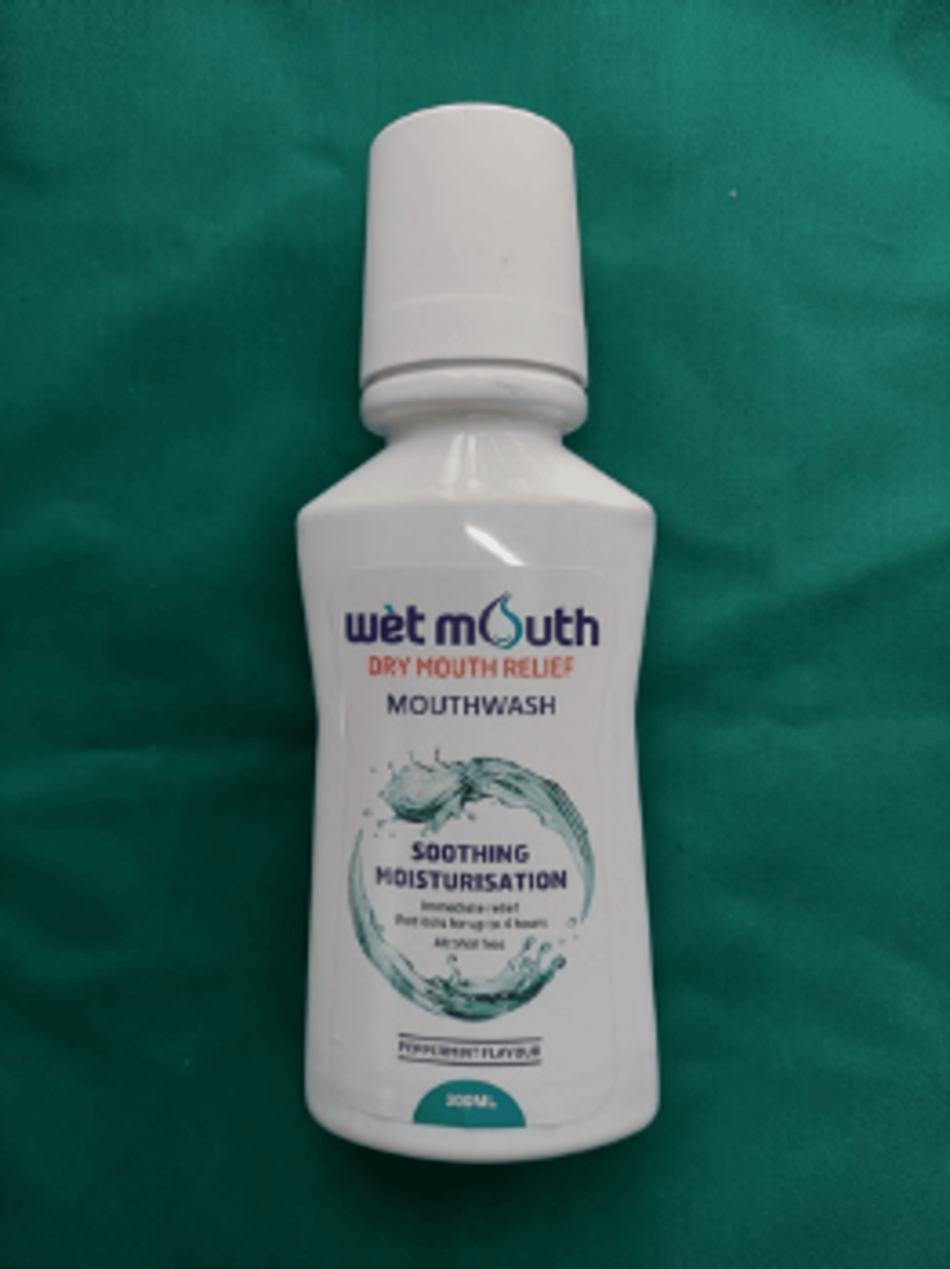
Artificial saliva.

Specimens were stored in artificial saliva (Wet Mouth, ICPA Health Products Ltd., Mumbai, India) at room temperature for 21 days (Figure 11).

Assessment of hydroxyproline release

**Figure 12:**
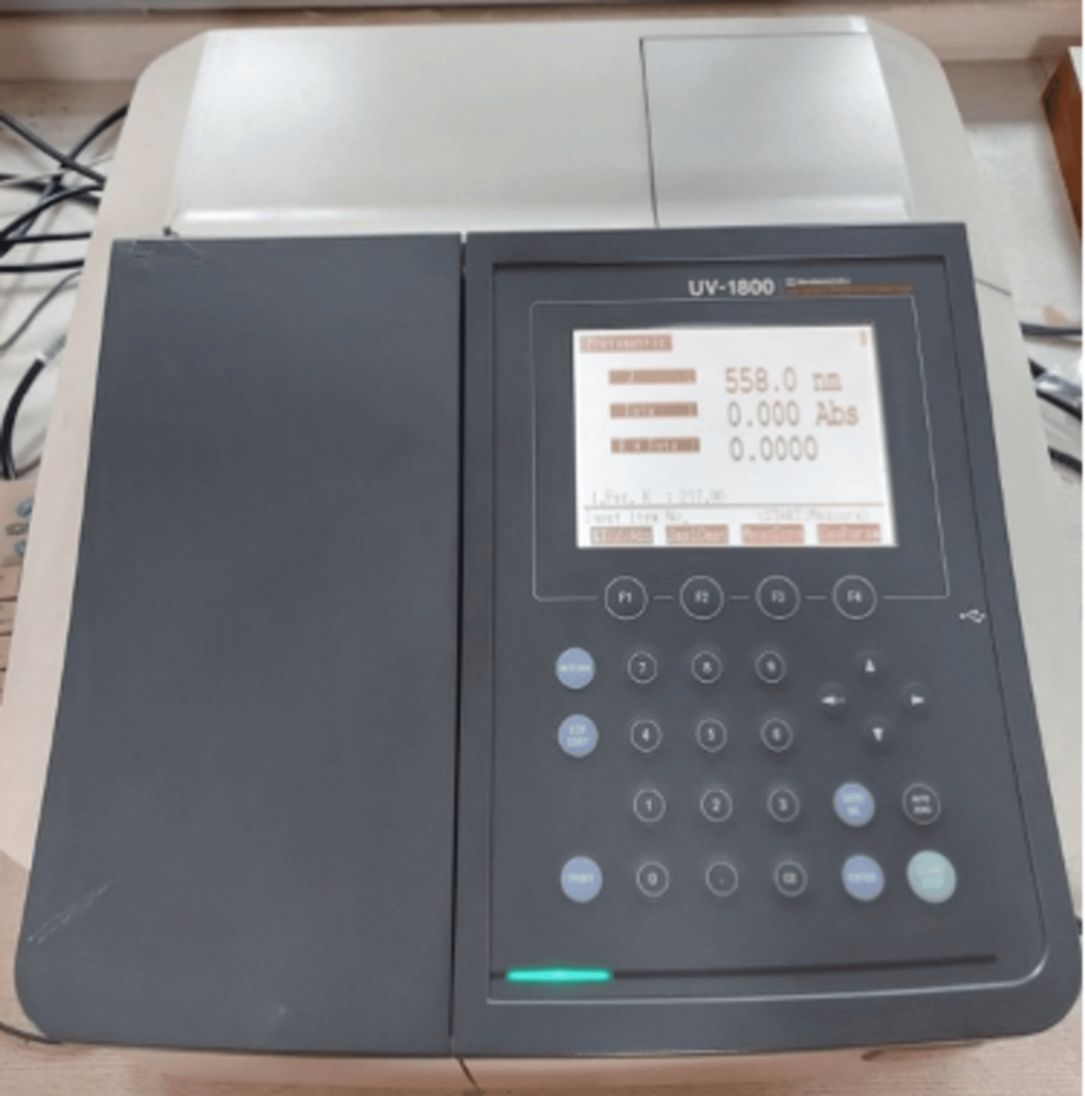
Spectrophotometer.

After 21 days, hydroxyproline (HYP) release, an indicator of collagen degradation, was measured from the storage medium using a HYP assay. Two hundred microliters of vortexed medium was placed into labeled ampules. Spectrophotometric analysis (UV-1800, Shimadzu Corporation, Kyoto, Japan ) was performed at 558 nm (Figure 12).

HYP concentration was calculated using the regression equation: Sa/Sv = C.

Where, Sa = amount of HYP (µg), from the standard curve; Sv = volume of sample added (µL); C = concentration of HYP in the sample.

Statistical analysis

**Figure 13:**
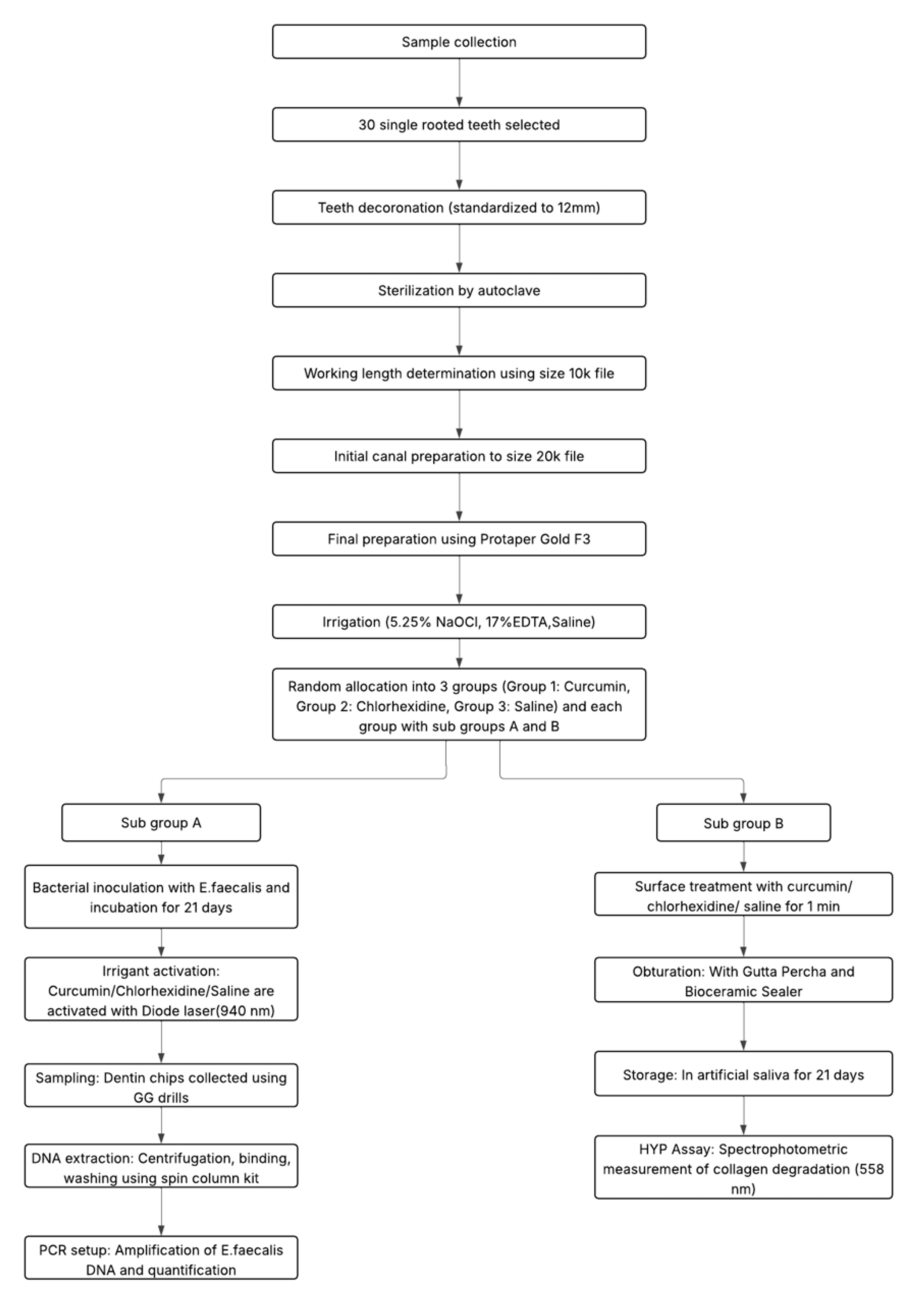
Flowchart of the methodology. EDTA: ethylenediaminetetraacetic acid; PCR: polymerase chain reaction; HYP: hydroxyproline.

Data were analyzed using IBM SPSS version 20.0 (IBM Corp., Armonk, NY). Mean values for cycle threshold (Ct), *E. faecalis* count (colony-forming units), and hydroxyproline release were calculated. Normality of data distribution was assessed using the Shapiro-Wilk test, and homogeneity of variances was evaluated using Levene’s test. As the data met parametric assumptions, intergroup comparisons were performed using one-way ANOVA, followed by Tukey’s post-hoc test for pairwise comparisons. A significance level of P < 0.05 was considered statistically significant (Figure 13).

## Results

Intergroup comparison of mean cycle threshold (Ct) values revealed a statistically significant difference (P < 0.05). Curcumin (Group 1A) exhibited the highest Ct value (mean ± SD = 38.20 ± 1.34), indicating the lowest bacterial load, followed by chlorhexidine (Group 2A: mean ± SD = 25.06 ± 3.11). The saline group (Group 3A) showed the lowest Ct values (Table 1).

**Table 1:** Intergroup comparison of mean cycle threshold values. Statistical analysis was performed using one-way ANOVA. * Statistical significance at P < 0.05.

Groups	Mean ± SD	F-value	P-value
Group 1A	38.20 ± 1.34	90.52	0.00*
Group 2A	25.06 ± 3.11		
Group 3A	15.54 ± 3.15		

Pairwise Ct value comparisons showed the smallest mean difference between curcumin and chlorhexidine (Group 1A vs. 2A, P = 0.00) and the largest difference between curcumin and saline (Group 1A vs. 3A, P = 0.00) (Table 2).

**Table 2:** Pairwise comparison of mean cycle threshold values. Post hoc analysis was performed using Tukey’s test. * Statistical significance at P < 0.05.

Pairwise comparison	Mean difference	P-value
Group 1A vs. Group 2A	13.14	0.00*
Group 1A vs. Group 3A	22.66	0.00*
Group 2A vs. Group 3A	9.52	0.00*

For *E. faecalis* counts, significant differences (P < 0.05) were observed across groups. Curcumin (Group 1A) had the lowest bacterial count (mean ± SD = 210.60 ± 46.11) (Table 3).

**Table 3:** Intergroup comparison of mean E. faecalis count (CFU). Statistical analysis was performed using one-way ANOVA. * Statistical significance at P < 0.05. CFU: colony-forming units.

Groups	Mean ± SD	F-value	P-value
Group 1A	210.60 ± 46.11	11.33	0.00*
Group 2A	1848.40 ± 1524.33		
Group 3A	57640.80 ± 37585.38		

The mean difference comparison of *E. faecalis* count shows the smallest difference between Groups 1A and 2A (P = 0.90), and the largest between Groups 1A and 3A (P = 0.00) (Table 4).

**Table 4:** Pairwise comparison of mean E. faecalis count (CFU). Post hoc analysis was performed using Tukey’s test. * Statistical significance at P < 0.05. CFU: colony-forming units.

Pairwise comparison	Mean difference	P-value
Group 1A vs. Group 2A	1637.80	0.90
Group 1A vs. Group 3A	57430.20	0.00*
Group 2A vs. Group 3A	55792.40	0.00*

Hydroxyproline release was lowest in the curcumin group (Group 1A: mean ± SD = 0.04 ± 0.02), while the highest value was seen in the control group (Group 3B: mean ± SD = 1.18 ± 0.28), with results statistically significant (P = 0.00) (Table 5).

**Table 5:** Intergroup comparison of mean hydroxyproline release. Statistical analysis was performed using one-way ANOVA. * Statistical significance at P < 0.05.

Groups	Mean ± SD	F-value	P-value
Group 1B	0.04 ± 0.02	72.16	0.00*
Group 2B	0.13 ± 0.02		
Group 3B	1.18 ± 0.28		

Pairwise comparisons of hydroxyproline release showed the least difference between Groups 1B and 2B (mean difference = 0.09; P = 0.38), followed by Groups 2B vs. 3B (mean difference = 1.04; P = 0.00). The highest difference was between Groups 1B and 3B (P = 0.00) (Table 6).

**Table 6:** Pairwise comparison of mean hydroxyproline release. Post hoc analysis was performed using Tukey’s test. * Statistical significance at P < 0.05.

Comparison group	Mean difference	P-value
Group 1B vs. Group 2B	0.09	0.38
Group 1B vs. Group 3B	1.13	0.00*
Group 2B vs. Group 3B	1.04	0.00*

## Discussion

The results of the present study demonstrated the superior effectiveness of curcumin over chlorhexidine and saline in terms of antimicrobial efficacy and preservation of collagen integrity, thereby rejecting the null hypothesis (Tables 1, 3).

Mechanical instrumentation is a critical component of successful root canal therapy, as it facilitates the removal of contaminated dentin and shapes the canals for effective irrigation and obturation. However, complex canal anatomy, such as lateral canals, isthmuses, and accessory branches, poses significant challenges to complete debridement. Moreover, bacteria residing in these areas can survive harsh environmental conditions and resist many conventional antibiotics [12].

*Enterococcus faecalis* is a particularly resilient Gram-positive anaerobe capable of withstanding such conditions, making it one of the most common causes of root canal failure and persistent infections. Its ability to compete with other microorganisms, invade dentinal tubules, resist nutrient deprivation, and form monospecies biofilms allows it to survive even under extreme conditions, including elevated temperatures [13,14]. Given these virulence traits, *E. faecalis* was chosen as the primary pathogen for investigation in this study.

In recent years, there has been a significant shift in oral microbiology from culture-based methods to molecular techniques. Among these, PCR amplification of 16S rDNA and other bacterial sequences has emerged as a highly specific and sensitive method for detecting both cultivable and non-cultivable species. Unlike endpoint PCR, quantitative real-time PCR (qPCR) not only detects specific bacterial genes but also allows quantification of bacterial load in clinical samples [15]. Sedgley et al. reported a significantly higher detection rate of *E. faecalis* using qPCR compared to culture techniques [16]. Hence, real-time PCR was employed in the current study to assess the antimicrobial efficacy of irrigants against *E. faecalis*.

Two irrigants (chlorhexidine and curcumin) were tested for both their antimicrobial action and collagen-stabilizing properties. Chlorhexidine is a well-established antimicrobial agent effective against a broad spectrum of microorganisms, including Gram-positive and Gram-negative bacteria, fungi, and yeasts. Its mechanism of action involves interaction between its positively charged molecules and the negatively charged phosphate groups on bacterial cell walls, leading to cell wall disruption and bacterial death [17].

In the present study, laser activation was employed to enhance irrigant efficacy. Laser activation improves canal decontamination by promoting cavitation and photoacoustic streaming, thereby enhancing irrigant penetration and debris removal [18]. Unlike chlorhexidine, curcumin acts as a natural photosensitizer in photodynamic therapy, producing reactive oxygen species (ROS) upon light activation. These ROS effectively destroy pathogens without damaging host tissues, which may explain the higher antimicrobial efficacy observed with curcumin (Tables 1, 3). This finding is supported by Diogo et al. [19], who reported that photoactivated sensitizers were more effective at disrupting *E. faecalis* biofilms than standard irrigants such as chlorhexidine and sodium hypochlorite. Additionally, photodynamic therapy is known to enhance the depth of irrigant penetration [20,21]. Sharifzadeh et al. (2023) [22] further reinforced this, reporting deeper canal penetration of curcumin when activated photodynamically, which may contribute to its superior antibacterial action.

The study also evaluated the collagen-stabilizing ability of the two irrigants. This aspect is particularly important with the current use of bioceramic sealers, which are favored for their bioactivity and sealing capabilities [22]. However, their high pH can compromise the structural integrity of dentin by disrupting the collagen network and increasing its permeability [23].

Curcumin and chlorhexidine are both known to act as cross-linking agents [24], potentially mitigating the detrimental effects of high-pH sealers on dentinal collagen. The present results align with this, as both agents demonstrated significantly lower hydroxyproline release than the control group (saline), indicating reduced collagen degradation (Table 5).

Collagen breakdown is mediated by proteolytic enzymes such as MMPs [25]. Curcumin inhibits this activity by binding to zinc ions (Zn²⁺), which are essential cofactors for MMP function [26,27]. Seseogullari-Dirihan et al. [28] observed substantial inhibition and complete inactivation of MMP-2 and MMP-9 following curcumin treatment of dentin. Similarly, chlorhexidine not only inhibits MMPs 2, 8, and 9 but also suppresses the activity of dentinal cysteine cathepsins B, K, and L, further preserving collagen matrix integrity [29].

Limitations

This study was conducted under in vitro conditions, which may not fully replicate the complex biological environment of the oral cavity. Variables such as the buffering capacity of dentinal fluid, host immune responses, and clinical challenges during irrigation and obturation were not considered. Additionally, the sample size was limited, and only a single bacterial species (*E. faecalis*) was evaluated. Further in vivo studies with larger sample sizes and polymicrobial biofilms are recommended to validate these findings. Additionally, curcumin, while promising, has practical limitations, including poor water solubility, photodegradation, a need for light activation, and potential for tooth and dentin discoloration. These factors must be considered when evaluating its clinical applicability, and further in vivo studies with larger, anatomically varied samples are necessary to validate the findings.

## Conclusions

Within the limitations of this in vitro study, curcumin demonstrated superior antimicrobial activity and collagen-stabilizing properties compared to chlorhexidine and saline. Its dual function as a natural photosensitizer and collagen cross-linker makes it a promising alternative irrigant in endodontic therapy. The reduced bacterial load and lower hydroxyproline release observed with curcumin suggest improved disinfection and enhanced preservation of dentinal collagen, potentially contributing to better long-term outcomes in root canal therapy. Its multifunctionality may also reduce the need for multiple irrigants and simplify clinical protocols. However, given its known limitations, such as poor solubility, photodegradation, and potential for staining, further in vivo studies and clinical trials are essential to evaluate its practical applicability and long-term effectiveness.
